# Resonance assignment of PsbP: an extrinsic protein from photosystem II of *Spinacia oleracea*

**DOI:** 10.1007/s12104-015-9606-2

**Published:** 2015-04-23

**Authors:** Adriana Rathner, Kousik Chandra, Petr Rathner, Michaela Horničáková, Judith Schlagnitweit, Jaroslava Kohoutová, Rüdiger Ettrich, Norbert Müller

**Affiliations:** Institute of Organic Chemistry, Johannes Kepler University, Altenbergerstraße 69, 4040 Linz, Austria; Faculty of Science, University of South Bohemia, České Budějovice, Czech Republic; Lohmann Animal Health, Heinz-Lohmann-Straße 4, 27472 Cuxhaven, Germany; Centre de RMN à très Hauts Champs, Institut des Sciences Analytiques, Université de Lyon, 5 Rue de la Doua, 69100 Villeurbanne, France; Centrum of Nanobiology and Structural Biology, Institute of Microbiology, Academy of Sciences of the Czech Republic, Nové Hrady, Czech Republic

**Keywords:** PsbP, Photosystem II, Oxygen evolving complex, Dynamic regions, Intrinsic disorder

## Abstract

PsbP (23 kDa) is an extrinsic eukaryotic protein of photosystem II found in the thylakoid membrane of higher plants and green algae. It has been proven to be indispensable for proper functioning of the oxygen evolving complex. By interaction with other extrinsic proteins (PsbQ, PsbO and PsbR), it modulates the concentration of two cofactors of the water splitting reaction, Ca^2+^ and Cl^−^. The crystallographic structure of PsbP from *Spinacia oleracea* lacks the N-terminal part as well as two inner regions which were modelled as loops. Those unresolved parts are believed to be functionally crucial for the binding of PsbP to the thylakoid membrane. In this NMR study we report ^1^H, ^15^N and ^13^C resonance assignments of the backbone and side chain atoms of the PsbP protein. Based on these data, an estimate of the secondary structure has been made. The structural motifs found fit the resolved parts of the crystallographic structure very well. In addition, the complete assignment set provides preliminary insight into the dynamic regions.

## Biological context

Photosystem II is a multi-protein, -lipid, and -pigment complex, which spans the thylakoid membrane of all photosynthetic organisms. Its protein fraction consists of two major parts, an intrinsic cluster of proteins and a set of extrinsic, “accessory”, proteins. While the intrinsic proteins are highly conserved among the photosynthetic species, the extrinsic proteins have evolved, probably from their homologous cyanobacterial precursors, as adaptations to the different photosynthetic apparatus in eukaryotes as compared to prokaryotes (Shen et al. 1998; Enami et al. 2005).

The PsbP protein is located on the lumenal side of thylakoids, in the oxygen evolving complex (OEC) of photosystem II, which is the site of the water-splitting reaction yielding molecular oxygen. PsbP is part of a barrier of extrinsic proteins, which surround the reaction centre with the Mn_4_CaO_5_ cluster at the lumenal thylakoid surface.

In total four extrinsic proteins have been found in most higher plants: PsbO (30 kDa), PsbP (23 kDa), PsbQ (16.5 kDa) and PsbR (10 kDa). The biggest of these, PsbO, is conserved across the all photosynthetic phyla, unlike the remaining three extrinsic proteins. The exact binding topology and interactions of these proteins still remain unclear, but recently more experimental data have yielded a clearer picture of the assembly of the entire OEC (Bricker et al. 2012; Järvi et al. 2013; Ido et al. 2014; Nishimura et al. 2014; Mummadisetti et al. 2014).

One known function of PsbP is controlling the concentrations of two co-factors of water oxidation—Ca^2+^ and Cl^−^ (Popelkova and Yocum 2007). Binding of PsbP to the thylakoid membrane induces structural changes, which are necessary for stable oxygen production during photosynthesis. The N-terminal segment of PsbP is indispensable for this conformational change to occur. When the PsbP protein is deprived of the 15 amino acids at the N-terminus, it is no longer capable of changing the topology of the membrane and the oxygen production decreases dramatically. In this case, it has been shown that PsbQ can compensate such a defect in the PsbP protein and helps to restore the levels of oxygen being released (Ifuku et al. 2011; Kakiuchi et al. 2012; Tomita et al. 2009). Preliminary studies in our group have provided the first solution structure of PsbQ. (Horničáková et al. 2011; Rathner 2013).

**Fig. 1:**
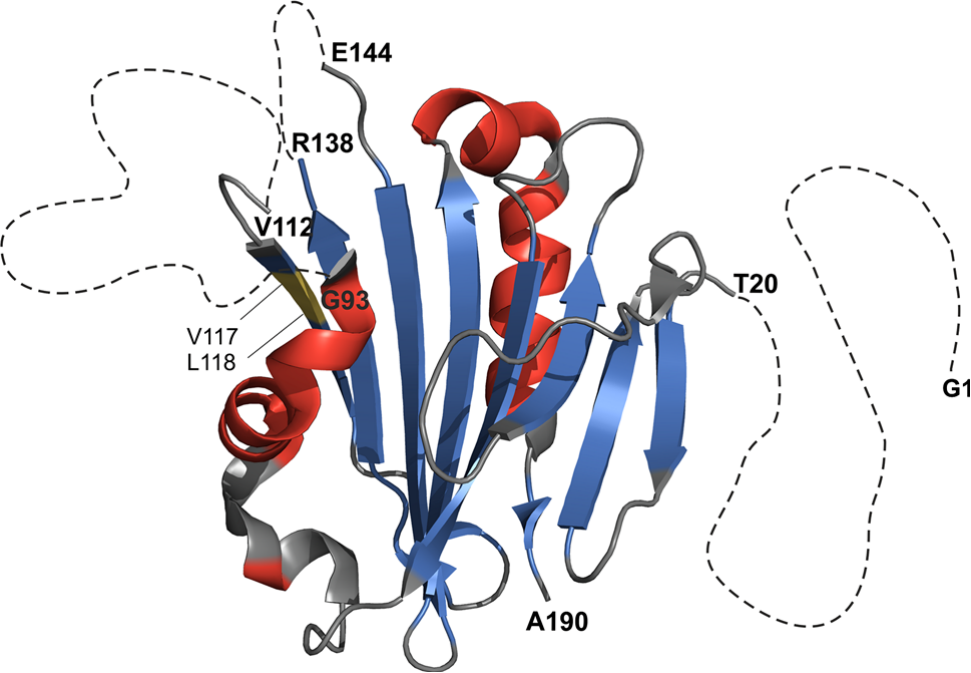
Cartoon representation of the X-ray crystallographic structure of PsbP (PDB ID: 2VU4) (Kopecký et al. 2012). The inner four stranded anti-parallel β-sheet core is surrounded by five short α-helices. The missing segments, labeled at the adjacent residues, are represented as *dotted lines*. The residues G1, V117 and L118, which are highlighted as well, could not be assigned by NMR. Numbering includes the four amino acids remaining from the His tag linker. The image was created using PyMOL (The PyMOL Molecular Graphics System, version 1.5.0.4 Schrödinger, LLC) from PDB ID: 2VU4

The three dimensional structure of PsbP from *Spinacia oleracea* has been resolved recently by X-ray crystallography at high resolution (1.98 Å) (Kopecký et al. 2012; Kohoutová et al. 2009), see Fig. 1. The resolved part of the structure compares very well with an earlier structure of PsbP from *Nicotiana tabacum* (Ifuku et al. 2004). The electron density was not resolved in the N-terminal residues (1–19) and in two internal sections (res. 94–111 and 139–143), although the crystal did not contain any degradation products. Additional data from Raman spectroscopy and molecular dynamics simulation suggested a dynamic nature for these regions. The N-terminus was modeled to contain a β-sheet element and the two unresolved internal regions to form two loops. Intrinsic disorder of the N-terminal part would be in accordance with its suggested function (Tomita et al. 2009; Ido et al. 2012).

In this paper we present the first NMR resonance assignment of PsbP as a foundation for further solution NMR studies of this protein, which will ultimately include interaction experiments with co-factors and other Psb proteins.

## Methods and experiments

### Protein expression and purification

The expression protocol followed Kohoutová et al. (2009) with some modifications. The gene coding for PsbP was included in the JR3133 plasmid, which also includes a His_6_ tag attached to the N-terminus of the protein. The transformed *E. coli* BL21DE3 cells were grown in 25 ml of LB medium (Kanamycin, 30 µg/ml) as an overnight culture. After 12 h of cultivation at 37 °C, the culture was diluted 1:100 and used to inoculate 1L of fresh LB medium. When the optical cell density reached 1 (after 2–3 h of cultivation at 37 °C with stirring at 150 rpm), the culture was centrifuged (45 min, 4000*g*, 30 °C). The pellet was resuspended in the corresponding amount of M9 medium containing (98 %) ^15^N ammonium sulfate (Sigma Aldrich) for the singly labeled protein and—in the case of doubly labeled protein production—(99 %) universally ^13^C labeled glucose (Sigma Aldrich). In both cases, the minimal medium was spiked with 1 ml of vitamin mixture (BME vitamins 100× solution). The cells were grown for 1 h at 37 °C and then induced with 1 mM IPTG. After induction, the cultivation temperature was lowered to 28 °C in order to prevent the undesirable formation of inclusion bodies. The cells were afterwards incubated overnight extending the total incubation time to 24 h, where an OD of 3 was achieved.

The harvested pellets were resuspended in 50 ml of ice-cold phosphate buffer (20 mM KH_2_PO_4_, 500 mM NaCl, 20 mM imidazole, 1 mM AEBSF, pH 7.4) and repeatedly sonicated on ice to release the soluble protein fraction. The lysate was filtered through a GVS filter (pore size 0.22 µm) to remove insoluble cell debris.

Since the protein included an N-terminal His_6_ tag, the first purification step was affinity chromatography using a Ni-Sepharose High Performance (GE Healthcare) column charged with NiSO_4_ and equilibrated with the binding buffer A (20 mM KH_2_PO_4_, 500 mM NaCl, 20 mM imidazole, pH 7.4). The lysate was applied to the column and immediately after the elution of the flow-through volume, His_6_-PsbP was eluted by a linear gradient of elution buffer A (20 mM KH_2_PO_4_, 500 mM NaCl, 500 mM imidazole, pH 7.4). The His-PsbP containing eluates were combined and concentrated with Amicon Ultra-15 filters (cut-off >10 kDa) and swapped into binding buffer B (20 mM Bis Tris, 1 mM EDTA, pH 6.0). The protein mixture was loaded onto a cation-exchange SP Sepharose Fast Flow column (GE Healthcare), which was washed with the binding buffer B. The elution was achieved with a linear gradient of elution buffer B (20 mM Bis Tris, 1 mM EDTA, 1 M NaCl, pH 6.0).

The eluted His_6_-PsbP was concentrated to 2.3 mg/ml in 11 ml and swapped into the tag cleavage buffer (20 mM Bis Tris, 100 mM NaCl, pH 6.0). The His_6_ tag was cleaved off by incubation with human plasma thrombin (Sigma Aldrich, 1.5 U per 1 mg of protein) for 3 h at 25 °C. The enzymatic digestion was quenched by addition of protease inhibitors (AEBSF). To remove the cleaved tag and the protease, size exclusion chromatography was employed as the last purification step. The mixture was loaded onto a Superdex 75 Prep Grade column (GE Healthcare) and pure PsbP was eluted with the running buffer (20 mM Bis Tris, 200 mM NaCl, pH 6.0) under constant flow of 0.3 ml/min after 3 h. Four additional amino acids (GSHM) from the His_6_ tag linker/cleavage site remained at the N-terminus of a final PsbP sample, bringing the total amino acid count to 190. The numbering used in this paper refers to the sequence of this recombinant protein.

### NMR experiments

All spectra were recorded on a 700 MHz Bruker Avance III spectrometer equipped with a TCI cryoprobe. The uniformly ^15^N, ^13^C labeled PsbP samples were exchanged into the NMR buffer (20 mM Bis Tris, 1 mM EDTA, 0.05 mM NaN_3_, pH 6.0, 10 % D_2_O) to a final concentration of 500 µM and transferred into 5 mm Shigemi tubes. The cleaving off of the N-terminal part could be prevented by adding a protease inhibitors cocktail (complete Mini, EDTA free from Roche Diagnostics) to each NMR sample (concentration of 12 tablets per 100 ml). This proved essential in order to prevent sample degradation over the long measurement periods at elevated temperature (samples remained stable at 40 °C for typically 21 days).

For the backbone assignment, the following set of spectra was recorded: ^1^H–^15^N HSQC, ^1^H–^13^C HSQC, HNCO, HNCACB, CBCA(CO)NH (Grzesiek and Bax 1992), HNHA (Vuister and Bax 1993), ^1^H–^15^N HSQC (Palmer et al. 1991). In order to achieve assignment of the aliphatic side chains, additional spectra were obtained: HCCH–COSY (Kay et al. 1993), H(CCO)NH, C(CO)NH (Montelione et al. 1992) and hCCH–TOCSY (Kay et al. 1993). All NMR spectra were processed using Bruker Topspin 3.1 or 3.2 software. The resonance assignment was carried out manually in the CARA (Keller 2004) and Sparky programs (Goddard and Kneller 2000). Secondary structure was analyzed using the online version of the Talos N program (Shen and Bax 2013), where the torsion angles φ, ψ and χ are estimated from experimental backbone chemical shifts (HN, H_α_, C_α_, C_β_, CO, N).

## Assignments and data deposition

### Assignments

**Fig. 2:**
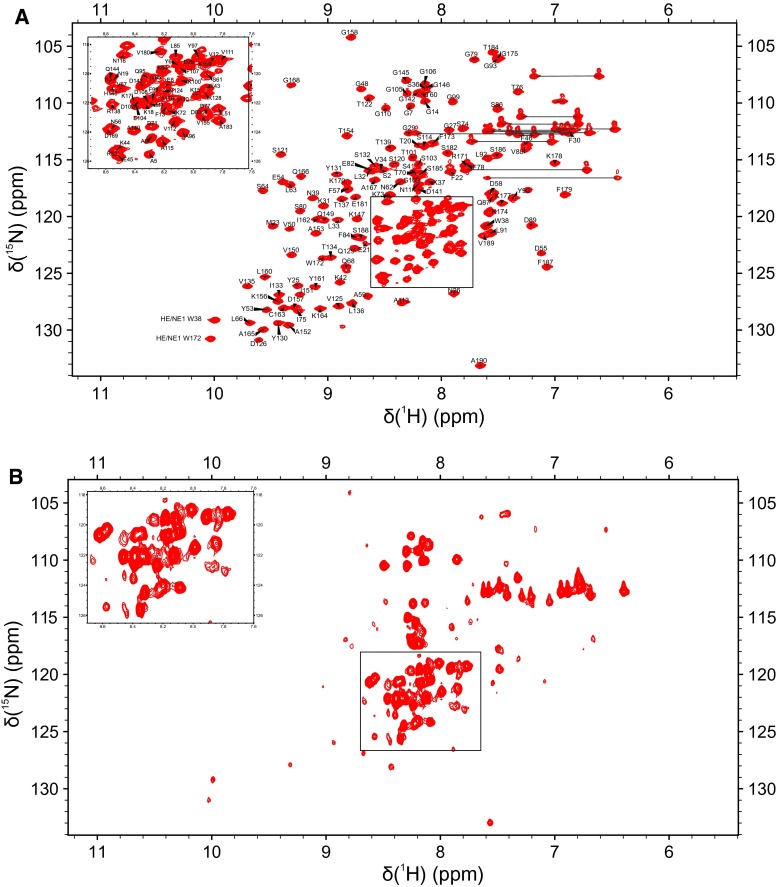
**a** ^1^H–^15^N HSQC spectrum of 500 µM ^15^N, ^13^C PsbP at 313 K (20 mM Bis Tris, 1 mM EDTA, 0.05 mM NaN_3_, pH 6.0, 10 % D_2_O, PI). The cross peaks are labeled with the respective residue types and numbers in the full recombinant protein sequence. The side chain NH peaks from the two tryptophan residues are labeled as well. Side chain NH_2_ peaks of glutamine and asparagine residues are connected by *thin horizontal lines*. **b** ^1^H–^15^N HSQC of PsbP recorded at 293 K under otherwise identical conditions

First NMR experiments were run at 293 K due to the low stability of the protein sample. However, ^1^H–^15^N HSQC as well as preliminary triple resonance experiments were hampered by high signal overlap (see Fig. 2) and severe broadening of some resonances, which precluded any significant number of assignments. To overcome this problem, all NMR experiments for assignment were then recorded at elevated temperature of 313 K after optimizing sample stability by a protease-inhibitor stabilized preparation (see preceding Section). This temperature increase led to a drastic improvement of resolution through narrowing of many broad lines presumably from regions of protein subject to internal dynamics. When the sample is cooled down to 293 K again, identical poorly resolved spectra are obtained implicating that the broadening effects are reversible and due to the dynamic properties of the protein. Preliminary diffusion experiments at 293 and 313 K show that the diffusion coefficient measured respectively, increases only by a factor of 1.13 (the diffusion coefficients were corrected for the change in water viscosity at given temperatures). This indicates that there is no significant change in the aggregation state upon temperature increase. The spectral dispersion observed at high temperature is characteristic of a well-folded protein and already upon visual inspection a high β-sheet content of PsbP can be recognized. With the spectra recorded at 313 K, 96 % of the backbone residues were assigned sequence-specifically (Fig. 2). Only, G1, V117, L118 and the amide nitrogen atoms of the 8 prolines could not be assigned. Assignment of side-chain resonances was accomplished to 81 %. Thus in total, 87 % of all resonances were assigned. This assignment enables us to proceed with the resolving of the solution structure of PsbP and to provide a basis for interaction studies between the Psb proteins. The assigned resonances have been deposited in the Biological Magnetic Resonance Data Bank under the Accession number 25379.

The central framed regions are magnified on the left sides of the spectra for better comparison. The spectra were recorded with two scans per increment and 2048 × 256 data points in the ^1^H and ^15^N dimensions, respectively.

### Secondary structure

**Fig. 3:**
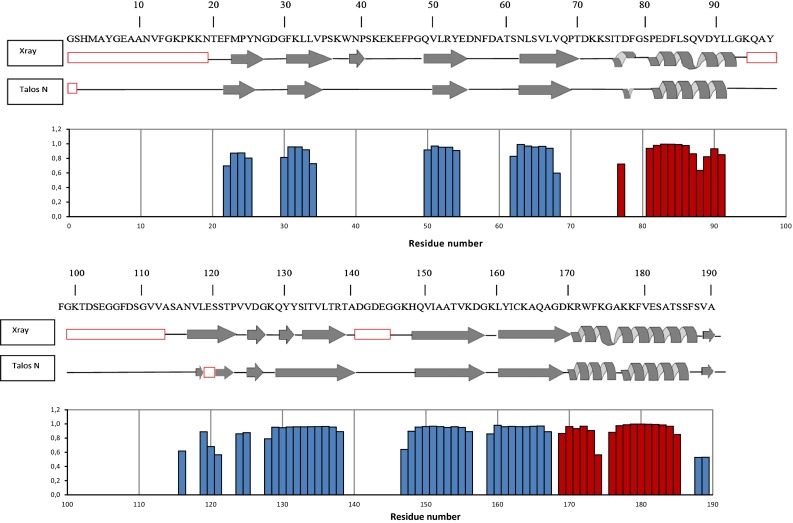
Secondary structure of PsbP. Secondary structure obtained from the X-ray crystallographic structure (PDB ID: 2VU4) and Talos N prediction are compared. The *bottom graph* shows the probability of the secondary structure elements calculated by Talos N. *Blue bars* indicate predicted β-sheet, while *red* is used for α-helices. Residues unresolved by X-ray or unassigned in NMR spectra are designated by *red rectangles*. Note that the numbering of the residues corresponds to the full recombinant protein sequence after tag cleavage, which compared to the wild type contains four additional amino acids (GSHM) at the N-terminus

Chemical shift based analysis of secondary structure elements was performed using the online platform version of Talos N (Shen and Bax 2013). The respective propensities of the three main secondary structure categories—α-helix, β-sheet and random coil were compared with the available crystallographic structure of PsbP (PDB ID: 2VU4) (Kopecký et al. 2012) as represented in Fig. 3. The juxtapositioning yielded almost perfect agreement within the crystallographically well-resolved β-sheet core and α-helices. From the NMR chemical shifts the secondary structures of the segments which were not deducible from the crystallographic electron densities, were estimated to be random coil in solution by Talos N. These results are corroborated by experimental NMR data at lower temperatures (293 K), where only resonances of these flexible regions appear well resolved in ^1^H–^15^N HSQC. Moreover no evidence for α-helix or β-sheet patterns in those particular regions (residues 1–19, 94–111 and 139–143) was found in ^1^H–^15^N NOESY–HSQC. The absence of peaks for V117 and L118 may be related to the dynamics of the nearby flexible loop. These findings confirm that under the above-mentioned conditions, the NMR sample contains a largely well-folded PsbP protein, which is well suitable for further detailed structural investigations by NMR, especially of its dynamic properties. The ultimate target of this ongoing research is the elucidation of the interaction network of PsbP with other Psb proteins, in particular PsbQ, PsbO and PsbR as well as their contacts to the thylakoid and intrinsic parts of photosystem II.

## Abbreviations

AEBSF: 4-(2-Aminoethyl) benzenesulfonyl fluoride hydrochloride
BME: Eagle’s basal medium
EDTA: Ethylenediaminetetraacetic acid
IPTG: Isopropyl β-d-1-thiogalactopyranoside
LB: Lysogeny broth
OD: Optical density
OEC: Oxygen evolving complex
PI: Protease inhibitors
